# The relationship between the presence and site of cancer, an inflammation-based prognostic score and biochemical parameters. Initial results of the Glasgow Inflammation Outcome Study

**DOI:** 10.1038/sj.bjc.6605855

**Published:** 2010-08-17

**Authors:** M J Proctor, D Talwar, S M Balmar, D S J O'Reilly, A K Foulis, P G Horgan, D S Morrison, D C McMillan

**Affiliations:** 1 University Department of Surgery, Faculty of Medicine-University of Glasgow, Royal Infirmary, Glasgow G31 2ER, UK; 2 Department of Clinical Biochemistry, Royal Infirmary, Glasgow G31 2ER, UK; 3 Department of Clinical Pathology, Royal Infirmary, Glasgow G31 2ER, UK; 4 University Department of Public Health and Health Policy, Faculty of Medicine-University of Glasgow, Glasgow G12 8QQ, UK

**Keywords:** C-reactive protein, albumin, adjusted calcium, liver function tests

## Abstract

**Background:**

Cancer incidence is increasing in the United Kingdom, as well as on a global basis. Biochemical parameters, such as C-reactive protein and albumin (combined to form the modified Glasgow Prognostic Score, mGPS), alkaline phosphatase (Alk phos), *γ*-glutamyl transferase (GGT) and serum calcium have been reported to be associated with cancer and non-cancer mortality. Therefore, to definitively examine the interrelationships between the above biochemical parameters, the mGPS and the presence of cancer, the Glasgow Inflammation Outcome Study was undertaken. The aim of this initial study was to examine the effect of cancer on markers of systemic inflammation induced by the liver (mGPS) and on levels of routine biochemical parameters.

**Methods:**

Patients (*n*=223 303) who had a single incidental sample taken for C-reactive protein, albumin, calcium and serum liver function tests where available, between 2000 and 2008 were studied. Those with a pathological diagnosis of cancer (*n*=22 715) were identified. The mGPS was constructed and liver function tests classified in accordance with the local reference ranges.

**Results:**

Patients with cancer had higher C-reactive protein and lower albumin levels (and thus a higher mGPS), higher adjusted calcium, Alk phos and GGT levels, but lower aspartate transaminase (AST) and alanine transaminase (ALT) levels (all *P*<0.001). The strongest associations (Spearman's correlation ⩾0.3) in both the non-cancer and cancer groups were found between albumin, C-reactive protein and Alk phos, AST and ALT, AST and GGT and ALT and GGT (all *P*<0.001). On multivariate analysis, the associations with the presence of cancer remained with age, deprivation, C-reactive protein, albumin, adjusted calcium, Alk phos and GGT (all *P*<0.01). Patients following a diagnosis of cancer had lower albumin levels and thus higher mGPS (all *P*<0.001). Also, post-diagnosis patients were more likely to have lower adjusted calcium, bilirubin, Alk Phos, AST, ALT and GGT levels (all *P*<0.05). When the cancer diagnoses were ranked from those with the lowest proportion of mGPS 1 or 2 to those with the highest, the percentage of cases with a mGPS of 1 or 2 ranged from 21% in breast cancer to 46% in prostate cancer and to 68% in pulmonary cancer. Compared with breast cancer the mGPS was significantly higher in those diagnosed with dermatological, bladder, endocrinological, gynaecological, prostate, musculoskeletal, gastroesophageal, haematological, renal, colorectal, head and neck, pancreaticobiliary and pulmonary cancers (all *P*<0.001).

**Conclusion:**

The results of the present study indicate that the systemic inflammatory response is common in a large patient cohort, increased by the presence of cancer and associated with the perturbation of a number of biochemical parameters previously reported to be associated with mortality. There is a striking parallel between the proportions of cases with a mGPS of 1 or 2 and reported survival rates in these tumours.

Cancer incidence is increasing in the United Kingdom, as well as on a global basis (Boyle and Levin, 2008). Over one in three people in the United Kingdom will develop cancer during their lifetime with around 150 000 people dying each year as a consequence (Cancer Research UK, 2007, 2008). Such a burden of disease accounts for a significant proportion of annual healthcare spending in the United Kingdom, the United States and worldwide (Bosanquet and Sikora, 2004; Boyle and Levin, 2008).

In the West of Scotland there has been a longstanding interest in the role of the systemic inflammatory response in determining outcome in cancer (McMillan, 2008, 2009; Roxburgh and McMillan, 2010). Although it is recognized that the development of cancer has a genetic basis, there is increasing evidence that host inflammatory responses have a pivotal role in the development and progression of cancer (Balkwill and Mantovani, 2001; Coussens and Werb, 2002; Mantovani *et al*, 2008; McDonald *et al*, 2009; Colotta *et al*, 2009). This is consistent with the hypothesis that chronic exposure to inflammatory processes, either through infection, social conditions or lifestyle, enhances the ageing process (Finch and Crimmins, 2004).

Recently, the presence of a systemic inflammatory response, as evidenced by an elevated C-reactive protein concentration sampled incidentally in a large hospital-based cohort, was associated with a shorter duration of cancer and non-cancer survival (Marsik *et al*, 2008). Goldwasser and Feldman (1997) reported a similar relationship in a systematic review of the prognostic value of albumin. These acute phase proteins, produced exclusively in the liver, have been shown to be a major factor in the progressive nutritional and functional decline of patients with cancer (McMillan, 2008). Also, C-reactive protein and albumin concentrations have been combined to form the modified Glasgow Prognostic Score (mGPS) and have been reported to be independently associated with reduced survival in patients with a variety of operable (Roxburgh and McMillan, 2010) and inoperable cancers (McMillan, 2009).

It is also of interest that liver function tests, such as bilirubin (Temme *et al*, 2001), alkaline phosphatase (Alk phos) (Tonelli *et al*, 2009) and *γ*-glutamyl transferase (GGT) (Kazemi-Shirazi *et al*, 2007), as well as serum calcium (Leifsson and Ahren, 1996), have also been reported to predict cancer and non-cancer outcomes. There is also some evidence that liver function tests are altered as part of the systemic inflammatory response in patients with lung and gastrointestinal cancer (Brown *et al*, 2007; Roxburgh *et al*, 2009).

Therefore, to definitively examine the interrelationships between the above biochemical parameters, the mGPS and the presence of cancer the Glasgow Inflammation Outcome Study was undertaken. The aim of this initial study was to examine the effect of cancer on markers of systemic inflammation induced by the liver (mGPS) and on levels of routine biochemical parameters (bilirubin, Alk phos, aspartate transaminase (AST), alanine transaminase (ALT), GGT and adjusted calcium).

## Materials and methods

### Study design

This cohort includes patients who have had a single sample taken for C-reactive protein, albumin and calcium between the first of January 2000 and the first of November 2008 in the North of Glasgow. The samples were taken incidentally and if more than one set of measurements were available for a given patient then only the initial set was used. Where available, serum liver function tests, including bilirubin, Alk phos, AST, ALT and GGT were recorded. Patients were excluded if they did not have a complete set of identifying details (name, gender, date of birth and hospital number).

All patient records fulfilling these criteria were then cross referenced with the local pathology database to identify those in whom a tissue diagnosis of cancer had been made either before or after their initial blood sample. Systematized Nomenclature of Medicine – Clinical Terms (SNOMED CT) codes, which share certain codes with the International Classification of Diseases, contained in the pathology database were used to identify the site and type of tumour in those with a tissue diagnosis of cancer. Patients in the cohort were then grouped into those with a pathological diagnosis of cancer and those without. The cancer groups were then grouped into those patients with a clear primary cancer diagnosis, those with multiple tumours or those with no clear indication of the primary tumour site. For the purpose of analysis, cancers were grouped according to tumour site; bladder, breast, colorectal, dermatological, endocrinological, gastroesophageal, gynaecological, haematological, head and neck, hepatocellular, musculoskeletal and soft tissue, pancreaticobiliary, pulmonary, prostatic, renal and testicular.

The study was approved by the Research Ethics Committee, North Glasgow NHS Trust.

### Methods

Patients with routine laboratory measurements of C-reactive protein, albumin and calcium were obtained by systematically searching the North Glasgow biochemical database system. The limit of detection of C-reactive protein was a concentration of less than 5 mg l^−1^. The mGPS was constructed as follows; patients with both an elevated C-reactive protein (>10 mg l^−1^) and hypoalbuminaemia (<35 g l^−1^) were allocated a score of 2; patients in whom C-reactive protein was elevated (>10 mg l^−1^) were allocated a score of 1 and those with both a normal C-reactive protein and albumin were allocated a score of 0 (McMillan *et al*, 2007). The rationale and basis of the mGPS has been previously described (McMillan, 2008). Serum C-reactive protein, albumin and liver function tests, including bilirubin, Alk phos, AST, ALT and GGT, as well as calcium adjusted for albumin (Ashby *et al*, 1986), were classified in accordance with the NHS Greater Glasgow and Clyde reference ranges (shown in Table 2). These analytes were measured using identical standard operating procedures on identical automated platforms using reagents from the same manufacturer (Abbot diagnostics). Analysis was carried out in three linked laboratories in the North of Glasgow that take part in external quality assurance schemes for routine biochemical analysis. All samples were refrigerated, as stable for a number of days in these conditions, and analyzed within 6 h.

Deprivation was measured with the Scottish Index of Multiple Deprivation 2006. The Scottish Index of Multiple Deprivation 2006 classification of deprivation is based on an individual's postcode and is derived from the measurements of 37 indicators across seven domains including income, employment, education, housing, health, crime and geographical access. The Scottish Index of Multiple Deprivation 2006 is the recommended method for the measurement of deprivation in Scotland by the Information Services Division on behalf of NHS Scotland and the Scottish Government Department of Health (Bishop *et al*, 2004).

SNOMED CT codes were used to identify the site and type of tumour in those with a tissue diagnosis of cancer. Each pathology sample had two SNOMED CT codes, one for type of tumour (morphology) and one for site of tumour (topography). Examples of SNOMED CT cancer morphology codes, of which there are over 400, include metastatic carcinoma (M80106), large-cell carcinoma (M80123), small-cell carcinoma (M80413-M80416), squamous-cell carcinoma (M80703-M80763), adenocarcinoma (M81402-M81406), cholangiocarcinoma (M81603), renal cell carcinoma (M83123-M83126), intraductal carcinoma (M85002-M85003), malignant melanoma (M87202-M87433), mesothelioma (M90501-M90503), lymphoma (M95903-M97003) and leukaemia (M98003-M99403). Examples of SNOMED CT topography codes include bladder (T74000-T74400), breast (T04000-T04400), colorectal (colon T67000-T67995, rectum T68000-T68200, anus T69000-T69200) dermatological (T02100-02870), endocrine (adrenal T93000-T93100, thyroid T96000-T97800), gastroesophageal (oesophagus T62000-T62910, stomach T63000-T63700), gynaecological (uterus with or without cervix T82000-T82900, uterus and ovaries T82920-T82922, cervix T83000-T83300, endometrium T84000-T85000, ovary T86920-T87800), haematological (bone marrow T06000-T06600, lymph node T08000-T09600), head and neck (upper respiratory tract T21000-T24920, mouth and salivary glands T51000-T55550, pharynx T60000-T61300), liver (T56000-T56020), musculoskeletal and soft tissue (skeletal bone T10000-T12720, skeletal muscle T13000) pancreaticobiliary (gallbladder T57000, bile duct T57600-T58500, pancreas and ampulla T58700-T59300) pulmonary(T26000-T29900), prostatic (T77000-T77350), renal (T71000-T72000) testicular (T78000-T78020). The combination of SNOMED CT morphology and topography codes were used to identify the tumour type and site.

### Statistics

The relationships between patient demographics, the presence of cancer, biochemical parameters, the mGPS, tumour site and other biochemical parameters were analzsed using the Pearson's *χ*^2^-test (linear by linear association). Spearman's rank correlation was used to measure the strength of the relationship between age and biochemical parameters and the interrelationships between biochemical parameters. The relationship between the presence of cancer and patient demographics and biochemical parameters was examined using binary logistic regression analysis; only patients who had a diagnosis of cancer made within 2 years following their blood test were included in this analysis. This was based on the premise that this group of patients were likely to have had an ongoing malignant process at the time of blood sampling. Analysis was performed using SPSS software (SPSS, Chicago, IL, USA).

## Results

In total 223 303 patients were studied. The majority, 144 900 (65%), were under 65 years of age. There were 120 115 (54%) females and 103 188 (46%) males. Of those patients with an identifiable postcode (86%), the majorities were from the Greater Glasgow area (88%). In this cohort, when measured by the Scottish Index of Multiple Deprivation 2006, 14% of cases were from affluent areas (least deprived quintile of the Scottish population) and 45% from deprived areas (most deprived quintile of the Scottish population).

The demographics of non-cancer and cancer patients are shown in Table 1. Patients with cancer, when compared with those without a diagnosis of cancer were older and more likely to be female (*P*<0.001). The cancer group had a higher proportion of cases from less deprived areas than those in the non-cancer group (*P*<0.001).

Of 22 715 with a diagnoses of cancer 19 476 (86%) had a single primary tumour, whereas 1299 (6%) had multiple malignancies. In 1940 (8%) cases SNOWMED CT codes gave no definitive indication of the primary site of malignancy. Examples of this include: 1497 cases were SNOWMED CT codes stated lung carcinoma, lung adenocarcinoma or lung metastatic carcinoma; 269 cases were SNOWMED CT codes stated liver carcinoma, liver adenocarcinoma or liver metastatic carcinoma; 209 cases had SNOWMED CT codes stated lymph node carcinoma, lymph node adenocarcinoma or lymph node metastatic carcinoma and 160 cases had SNOWMED CT codes stating metastatic carcinoma at other sites.

When the cancer group was categorized (*n*=22 715) into different tumour sites the following main groups were observed; bladder (*n*=939, 4%), breast (*n*=3849, 17%), colorectal (*n*=1899, 8%), dermatological (*n*=4907, 22%), endocrinological (*n*=303, 1%), gastroesophageal (*n*=1018, 5%), gynaecological (*n*=1136, 5%), haematological (*n*=1165, 5%), head and neck (*n*=610, 3%), hepatocellular (*n*=55, 0%), musculoskeletal and soft tissue (*n*=164, 1%), pancreaticobiliary (*n*=456, 2%), prostate (*n*=957, 4%), pulmonary (*n*=1393, 6%), renal (*n*=527, 2%) and testicular (*n*=97, 1%).

The patient biochemical parameters in the non-cancer and cancer groups are shown in Table 2. There were higher circulating concentrations of C-reactive protein and lower albumin levels (and thus a higher proportion of mGPS 1 or 2), in those patients with cancer (all *P*<0.001). Patients in the cancer group were also found to have lower AST and ALT levels, but higher adjusted calcium, Alk phos and GGT levels compared with the non-cancer cohort (all *P*<0.001).

The interrelationships between age and biochemical parameters in the non-cancer and cancer cohorts are shown in Tables 3 and 4. In the non-cancer cohort age was significantly associated with all the biochemical parameters (all *P*<0.001) (Table 3). Similarly, all biochemical parameters were significantly associated with each other (all *P*<0.001). In the non-cancer cohort the strongest associations (Spearman's correlation ⩾0.3) were between C-reactive protein and albumin, C-reactive protein and Alk phos, AST and ALT, AST and GGT and ALT and GGT.

There were similar interrelationships in the cancer cohort (Table 4) with the strongest associations (Spearman's correlation ⩾0.3) existing between C-reactive protein and albumin, C-reactive protein and Alk phos, bilirubin and AST, Alk phos and GGT, AST and ALT, AST and GGT and ALT and GGT.

The temporal relationships between the date of blood samples, biochemical parameters and the mGPS in the cancer group are shown in Table 5. To give a clearer indication of the alterations that occur in biochemical parameters surrounding the diagnosis of cancer, only those patients who were diagnosed within 2 years of their blood sample being analyzed were included in the analysis, pre-diagnosis (*n*=8083) and post diagnosis (*n*=5971). Compared with those patients who had pre-diagnosis levels, post-diagnosis patients were more likely to be female and affluent (all *P*<0.001). Patients following a diagnosis of cancer had lower albumin levels and thus higher mGPS (all *P*<0.001). Also, post-diagnosis patients were more likely to have lower adjusted calcium, bilirubin, Alk Phos, AST, ALT and GGT levels (all *P*<0.05).

The relationship between patient demographics, biochemical parameters and the absence (*n*=200 588) or presence (*n*=8083) of cancer within 2 year-pre-diagnosis group is shown in Table 6. On univariate analysis, those over 65 years of age and living in least deprived areas were more likely to have cancer (all *P*<0.001). Patients with an elevated C-reactive protein, adjusted calcium, Alk phos and GGT levels or low albumin levels were more likely to have cancer (all *P*<0.001). On multivariate analysis, these associations with the presence of cancer persisted in age, deprivation, C-reactive protein, albumin, adjusted calcium, Alk phos and GGT (all *P*<0.01).

The relationships between the mGPS and tumour site in the 2-year pre-diagnosis group is shown in cancers with more than 50 cases in Table 7. The cancers are ranked from those with the highest proportion of mGPS 0 to those with the lowest. The percentage of cases with a mGPS of 1 or 2 ranges from 21% in breast cancer, to 46% in prostate cancer and to 68% in pulmonary cancer. Compared with breast cancer the mGPS was significantly higher in dermatological, bladder, endocrinological, gynaecological, prostate, musculoskeletal, gastroesophageal, haematological, renal, colorectal, head and neck, pancreaticobiliary and pulmonary cancers (all *P*<0.001).

## Discussion

The initial results of the Glasgow Inflammation Outcome Study show that the presence of a systemic inflammatory response, as evidenced by an elevated C-reactive protein concentration (>10 mg l^−1^), was present in 40% and hypoalbuminaemia (<35 g l^−1^) was present in 14% of 223 303 patients who were incidentally sampled. Adjusted calcium was elevated in 2.2% (1.8% unadjusted), Alk phos was elevated in 12%, ALT was elevated in 11% and GGT was elevated in 26% of patients studied.

These results are consistent with previous studies including an Austrian cohort of approximately 280 000 patients, in whom 35% had an elevated C-reactive protein concentration (Marsik *et al*, 2008) and 13% had hypoalbuminaemia (Grimm *et al*, 2009). They also reported that 33% had an elevated GGT concentration (Kazemi-Shirazi *et al*, 2007). In a Swedish population-based cohort of 33 346 patients, 1% were found to have an elevated unadjusted serum calcium (Leifsson and Ahren, 1996). In a North American cohort of 18 835 patients approximately 9% had an elevated Alk phos (Tonelli *et al*, 2009). Another population-based cohort from the United States of 14 950 patients reported an elevated ALT level in 14% of patients (Ruhl and Everhart, 2009). Therefore, the results of the present study are similar to those previously reported in other large cohort studies.

In the present study there were more females in the cohort as a whole, more females in the cancer group and more females had measurements taken after cancer diagnosis. However, gender was not significantly associated with the presence of cancer. The present study shows that when compared with non-cancer, the presence of cancer was associated with a significantly higher proportion of cases with an elevated C-reactive protein (>10 mg l^−1^, 47 *vs* 39%) and lower albumin (<35 g l^−1^, 19 *vs* 14%) concentrations, higher adjusted calcium (>2.60 mmol l^−1^, 4 *vs* 2%), Alk phos (>280 U l^−1^, 16 *vs* 12%) and GGT (⩾40 U l^−1^ in females, ⩾70 U l^−1^ in males, 29 *vs* 25%) and lower ALT levels (⩾50 U l^−1^, 10 *vs* 12%). Furthermore, when samples taken following a diagnosis of cancer were compared with those taken before a diagnosis of cancer the proportion of cases with albumin (⩾35 g l^−1^, 74 *vs* 81%) adjusted calcium (>2.60 mmol l^−1^, 3 *vs* 5%), Alk phos (>280 U l^−1^, 15 *vs* 18%), ALT (⩾50 U l^−1^, 10 *vs* 11%). and GGT (⩾40 U l^−1^, in females and ⩾70 U l^−1^ in males 29 *vs* 31%) levels were all lower. When these biochemical parameters, together with bilirubin and ALT, were regressed against the presence of cancer, C-reactive protein, albumin, adjusted calcium, Alk phos and GGT were all shown to be independently associated with the presence of cancer. These results would suggest that the presence of cancer influences a number of biochemical parameters that have been previously associated with all cause mortality (Leifsson and Ahren, 1996; Kazemi-Shirazi *et al*, 2007; Marsik *et al*, 2008; Grimm *et al*, 2009, Tonelli *et al*, 2009).

The present study also showed that all these biochemical parameters were significantly associated. In particular, C-reactive protein was associated with albumin and Alk phos and GGT associated with AST and ALT in both cancer and non-cancer cohorts. Recently the combination of C-reactive protein and albumin, termed the mGPS, was shown to be associated in a similar manner in patients with lung cancer (Brown *et al*, 2007) and predict cancer-specific survival in a number of operable (Roxburgh and McMillan, 2010) and inoperable cancers (McMillan, 2009). These results raise the question of whether the above reported association of these biochemical parameters and all cause mortality are indeed independent of each of other. Further studies using this cohort will aim to address this question.

In the present study the proportions of mGPS 1 or 2 were greater following a diagnosis of cancer. This appears to be secondary to a reduction in albumin that may be secondary to a number of factors, including operative management, adjuvant oncological therapies or tumour progression, a pattern recognized in previous studies within specific tumour types (McMillan *et al*, 2003; Jamieson *et al*, 2005). It is interesting that in patients who had blood tests taken following a diagnosis of cancer, adjusted calcium, bilirubin and Alk phos, AST, ALT and GGT levels were all more likely to be within normal limits. The reasons for this are unclear but it is possible that removal or oncological treatment of the underlying malignant process in a proportion of these patients may be responsible.

In the present study it was also of interest to note that, before diagnosis, there were significant variations in the proportions of cases with a mGPS of 1 or 2. Indeed, the proportion of patients with an elevated mGPS varied from 21% in breast cancer, to 46% in prostate cancer and to 68% in pulmonary cancer. There is a striking similarity between the proportions of cases with a mGPS of 1 or 2 and reported survival rates in these tumours (Cancer Research UK, 2004). For example, the 5-year survival rate for breast cancer is 77% and the proportion of patients in the present study with a mGPS of 0 was 79%. In contrast the 5-year survival for lung cancer is 6% and the proportion of patients in the present study with a mGPS of 0 was 32%. These results may suggest that, in addition to the mGPS having prognostic value within tumour types (McMillan, 2009; Roxburgh and McMillan, 2010) it may also have prognostic value across tumour types. Further work is required to confirm this hypothesis.

The present cohort has a number of limitations. The patients were selected on the basis that measurements of C-reactive protein, albumin and calcium had been performed and were, therefore, not necessarily representative of all non-cancer and cancer patients treated in the North Glasgow area and, therefore, gives no meaningful information on cancer prevalence. Furthermore, sampling was incidental and not performed at a standard time during the course of the disease in the cancer group. It is also recognized that patients in both groups may have concurrent morbidity causing a rise in their C-reactive protein and derangement of their albumin and other biochemical parameters. As inclusion in the cancer cohort was dependent on a pathological diagnosis of cancer it is acknowledged that a small number in the non-cancer group may have had a clinical diagnosis of malignancy. To provide more detailed follow with regards to survival it is important to link the present data with patient outcomes.

In summary, the results of the present study indicate that the systemic inflammatory response is common in a large patient cohort, increased by the presence of cancer and associated with the perturbation of a number of biochemical parameters previously reported to be associated with mortality. Taken together with the myriad effects of the systemic inflammatory response on host metabolism (Gabay and Kushner, 1999) these results have a number of clinical implications particularly for clinical epidemiological studies. Future studies should incorporate a measure of the systemic inflammatory response, such as C-reactive protein or the mGPS, in the study design, analysis and interpretation. This is required to account for the likely confounding effect of this response on the other measured variables and their relationship with outcomes.

## Figures and Tables

**Table 1:** Patient demographics in the non-cancer and cancer groups in the Glasgow Inflammation Outcome Study

	**Non-cancer *n* (%) 200 588**	**Cancer *n* (%) 22 715**	***P*-value**
*Age (years)*			
<65	135 531 (67)	9369 (41)	
65–74	31 593 (16)	6412 (28)	
⩾75	33 464 (17)	6934 (31)	<0.001
			
*Sex*			
Male	93 313 (46)	9875 (43)	
Female	107 275 (54)	12 840 (57)	<0.001
			
*SIMD 2006*			
1 (Most deprived)	77 448 (45)	8844 (42)	
2	30 850 (18)	3872 (18)	
3	21 148 (12)	2771 (13)	
4	18 693 (11)	2578 (12)	
5 (Least deprived)	22 902 (14)	3233 (15)	<0.001

**Table 2:** The relationship between the presence of cancer, biochemical parameters and the mGPS in the Glasgow Inflammation Outcome Study

	**Non-cancer *n* (%) 200 588**	**Cancer *n* (%) 22 715**	***P*-value**
*C-reactive protein*			
⩽10 mg l^−1^	122 317 (61)	12 050 (53)	
>10 mg l^−1^	78 271 (39)	10 665 (47)	<0.001
			
*Albumin*			
<35 g l^−1^	27 515 (14)	4275 (19)	
⩾35 g l^−1^	173 073 (86)	18 440 (81)	<0.001
			
*mGPS*			
0	122 317 (61)	12 050 (53)	
1	56 357 (28)	7033 (31)	
2	21 914 (11)	3632 (16)	<0.001
			
*Adjusted calcium*			
<2.10 mmol l^−1^	4963 (3)	577 (2)	
2.10–2.60 mmol l^−1^	191 429 (95)	21 323 (94)	
>2.60 mmol l^−1^	4196 (2)	815 (4)	<0.001
			
*Bilirubin*			
<20 *μ*mol l^−1^	170 447 (88)	19 322 (88)	
⩾20 *μ*mol l^−1^	24 258 (12)	2593 (12)	0.008
			
*Alkaline phosphatase*			
<80 U l^−1^	28 168 (14)	2463 (11)	
80–280 U l^−1^	148 245 (74)	16 626 (73)	
>280 U l^−1^	23 954 (12)	3603 (16)	<0.001
			
*Aspartate transaminase*			
<40 U l^−1^	162 269 (84)	18 676 (86)	
⩾40 U l^−1^	30 531 (16)	3129 (14)	<0.001
			
*Alanine transaminase*			
<50 U l^−1^	144 468 (88)	15 872 (90)	
⩾50 U l^−1^	19 043 (12)	1740 (10)	<0.001
			
*γ-Glutamyl transferase*			
M<70 U l^−1^, F<40 U l^−1^	145 658 (75)	15 689 (71)	
M⩾70U l^−1^, F⩾40 U l^−1^	49 393 (25)	6325 (29)	<0.001

Abbreviation: mGPS=modified Glasgow Prognostic Score.

**Table 3:** The relationship between biochemical parameters in the non-cancer group of the Glasgow Inflammation Outcome Study

***n*=200 588**	**C-reactive protein**	**Albumin**	**Adjusted calcium**	**Bilirubin**	**Alkaline phosphatase**	**Aspartate transaminase**	**Alanine transaminase**	***γ*-Glutamyl transferase**
Age	0.199^***^	−0.286^***^	0.052^***^	0.099^***^	0.177^***^	0.078^***^	−0.047^***^	0.148^***^
C-reactive protein		−0.362^***^	−0.108^***^	0.167^***^	0.320^***^	0.103^***^	0.040^***^	0.193^***^
Albumin			−0.060^***^	0.040^***^	0.022^***^	0.009^***^	0.072^***^	−0.116^***^
Adjusted calcium				−0.077^***^	−0.147^***^	−0.081^***^	−0.028^***^	0.077^***^
Bilirubin					0.143^***^	0.297^***^	0.216^***^	0.169^***^
Alkaline phosphatase						0.225^***^	0.152^***^	0.243^***^
Aspartate transaminase							0.736^***^	0.437^***^
Alanine transaminase								0.550^***^

^***^P<0.001.

**Table 4:** The relationship between biochemical parameters in the cancer group of the Glasgow Inflammation Outcome Study

***n*=22 715**	**C-reactive protein**	**Albumin**	**Adjusted calcium**	**Bilirubin**	**Alkaline phosphatase**	**Aspartate transaminase**	**Alanine transaminase**	***γ*-Glutamyl transferase**
Age	0.103^***^	−0.203^***^	0.024^***^	0.127^***^	0.079^***^	0.020^**^	−0.162^***^	−0.062^***^
C-reactive protein		−0.439^***^	−0.078^***^	0.166^***^	0.308^***^	0.104^***^	0.034^***^	0.239^***^
Albumin			−0.086^***^	0.004	0.028^***^	0.007	0.073^***^	−0.123^***^
Adjusted calcium				−0.095^***^	−0.087^***^	−0.053^***^	−0.013	0.106^***^
Bilirubin					0.166^***^	0.307^***^	0.225^***^	0.177^***^
Alkaline phosphatase						0.253^***^	0.213^***^	0.346^***^
Aspartate transaminase							0.720^***^	0.403^***^
Alanine transaminase								0.505^***^

^***^P<0.001.

**Table 5:** The relationship between time of diagnosis, biochemical parameters and the mGPS in the cancer group of the Glasgow Inflammation Outcome Study

	**Pre-diagnosis *n* (%) 8083**	**Post-diagnosis *n* (%) 5971**	***P*-value**
*Age (years)*			
<65	3516 (43)	2690 (45)	
65–74	2413 (30)	1626 (27)	
⩾75	2154 (27)	1655 (28)	0.732
			
*Sex*			
Male	3806 (47)	2482 (42)	
Female	4277 (53)	3489 (58)	<0.001
			
*SIMD 2006*			
1 (Most deprived)	3042 (40)	2011 (37)	
2	1428 (19)	1003 (18)	
3	1080 (14)	802 (15)	
4	958 (12)	724 (13)	
5 (Least deprived)	1140 (15)	951 (17)	<0.001
			
*C-reactive protein*			
⩽10 mg l^−1^	4082 (51)	2963 (50)	
>10 mg l^−1^	4001 (49)	3008 (50)	0.304
			
*Albumin*			
<35 g l^−1^	1566 (19)	1545 (26)	
⩾35 g l^−1^	6517 (81)	4426 (74)	<0.001
			
*mGPS*			
0	4082 (50)	2963 (50)	
1	2666 (33)	1643 (27)	
2	1335 (17)	1365 (23)	<0.001
			
*Adjusted calcium*			
<2.10 mmol l^−1^	183 (2)	151 (3)	
2.10–2.60	7501 (93)	5614 (94)	
>2.60 mmol l^−1^	399 (5)	206 (3)	<0.001
			
*Bilirubin*			
<20 *μ*mol l^−1^	6844 (87)	5163 (90)	
⩾20 *μ*mol l^−1^	994 (13)	587 (10)	<0.001
			
*Alkaline phosphatase*			
<80 U l^−1^	788 (10)	743 (12)	
80–280 U l^−1^	5805 (72)	4324 (73)	
>280 U l^−1^	1482 (18)	898 (15)	0.003
			
*Aspartate transaminase*			
<40 U l^−1^	6614 (85)	4941 (86)	
⩾40 U l^−1^	1175 (15)	788 (14)	0.030
			
*Alanine transaminase*			
<50 U l^−1^	5729 (89)	4215 (90)	
⩾50 U l^−1^	734 (11)	450 (10)	0.004
			
*γ-Glutamyl transferase*			
M<70 U/l, F<40 U l^−1^	5440 (69)	4100 (71)	
M⩾70 U/l, F⩾40 U l^−1^	2432 (31)	1667 (29)	0.012

**Table 6:** The relationship between patient demographics, biochemical parameters and the presence of cancer in the Glasgow Inflammation Outcome Study

	**Univariate analysis**			**Multivariate analysis**		
	**OR**	**95% CI**	***P*-value**	**OR**	**95% CI**	***P*-value**
*Age*						
<65/	1		<0.001	1		<0.001
65–74	2.94	2.79–3.12	<0.001	2.60	2.46–2.75	<0.001
⩾75	2.48	2.35–2.62	<0.001	2.17	2.05–2.30	<0.001
						
*Sex*						
Male	1		0.317			
Female	0.98	0.94–1.02	0.317			
						
*SIMD*						
1 (Most deprived)	1		<0.001	1		<0.001
2	1.18	1.11–1.26	<0.001	1.16	1.09–1.24	<0.001
3	1.30	1.21–1.40	<0.001	1.32	1.22–1.42	<0.001
4	1.31	1.21–1.41	<0.001	1.36	1.26–1.46	<0.001
5 (Least deprived)	1.27	1.18–1.36	<0.001	1.27	1.18–1.36	<0.001
						
*C-reactive protein*						
⩽10 mg l^−1^	1		<0.001	1		<0.001
>10 mg l^−1^	1.53	1.47–1.60	<0.001	1.22	1.16–1.28	<0.001
						
*Albumin*						
<35 g l^−1^	1.51	1.43–1.60	<0.001	1.11	1.04–1.19	0.002
⩾35 g l^−1^	1		<0.001	1		0.002
						
*Adjusted calcium*						
<2.10 mmol l^−1^	0.94	0.81–1.09	0.425			
2.10–2.60 mmol l^−1^	1		<0.001			<0.001
>2.60 mmol l^−1^	2.43	2.18–2.70	<0.001	1.90	1.70–2.21	<0.001
						
*Bilirubin*						
<20 *μ*mol l^−1^	1		0.558			
⩾20 *μ*mol l^−1^	1.02	0.95–1.09	0.558			
						
*Alkaline phosphatase*						
<80 U l^−1^	0.71	0.66–0.77	<0.001	0.81	0.75–0.87	<0.001
80–280 U l^−1^	1		<0.001	1		<0.001
>280 U l^−1^	1.58	1.49–1.68	<0.001	1.30	1.22–1.39	<0.001
						
*Aspartate transaminase*						
<40 U l^−1^	1		0.075			
⩾40 U l^−1^	0.94	0.89–1.01	0.075			
						
*Alanine transaminase*						
<50 U l^−1^	1		0.477			
⩾50 U l^−1^	0.97	0.90–1.05	0.477			
						
*γ-Glutamyl transferase*						
M<70 U l^−1^, F<40 U l^−1^	1		<0.001	1		
M⩾70 U l^−1^, F⩾40 U l^−1^	1.32	1.26–1.38	<0.001	1.09	1.03–1.15	0.003

**Table 7:** The relationship between tumour site and an inflammation-based prognostic score (mGPS) in the Glasgow Inflammation Outcome Study

		**mGPS *n* (%)**			
**Tumour site**	**Total *N***	**0**	**1**	**2**	***P*-value^a^**
Breast	1199	950 (79)	207 (17)	42 (4)	
Dermatological	1126	693 (62)	310 (27)	123 (11)	<0.001
Bladder	296	174 (59)	76 (26)	46 (15)	<0.001
Endocrinological	145	84 (58)	41 (28)	20 (14)	<0.001
Gynaecological	197	108 (55)	60 (30)	29 (15)	<0.001
Prostate	267	145 (54)	91 (34)	31 (12)	<0.001
Musculoskeletal	78	41 (53)	15 (19)	22 (28)	<0.001
Gastroesophageal	503	249 (50)	162 (32)	92 (18)	<0.001
Haematological	499	245 (49)	157 (32)	97 (19)	<0.001
Renal	294	132 (45)	105 (36)	57 (19)	<0.001
Colorectal	784	342 (44)	250 (32)	192 (24)	<0.001
Head and neck	156	67 (43)	58 (37)	31 (20)	<0.001
Pancreaticobiliary	321	122 (38)	101 (31)	98 (31)	<0.001
Pulmonary	820	260 (32)	387 (47)	173 (21)	<0.001

Abbreviation: mGPS=modified Glasgow Prognostic Score.

a Compared with breast cancer.
